# Prenatal and infantile diagnosis of craniosynostosis in individuals with RASopathies

**DOI:** 10.1002/ajmg.a.63397

**Published:** 2023-09-29

**Authors:** Carolyn R. Serbinski, April Vanderwal, Sarah E. Chadwell, Ana Isabel Sanchez, Robert J. Hopkin, Robert B. Hufnagel, K. Nicole Weaver, Carlos E. Prada

**Affiliations:** 1 Division of Human Genetics, Cincinnati Children’s Hospital Medical Center, Cincinnati, Ohio, USA; 2 Division of Genetics, Genomics, and Metabolism, Ann & Robert H. Lurie Children’s Hospital of Chicago, Chicago, Illinois, USA; 3 Department of Genetics, Health Research Institute-Fundación Jiménez Díaz University Hospital, Universidad Autónoma de Madrid (IIS-FJD, UAM), Madrid, Spain; 4 Department of Pediatrics, University of Cincinnati College of Medicine, Cincinnati, Ohio, USA; 5 Ophthalmic Genetics and Visual Function Branch, National Eye Institute, National Institutes of Health, Bethesda, Maryland, USA; 6 Fundación Cardiovascular de Colombia, Bucaramanga, Santander, Colombia; 7 Department of Pediatrics, Feinberg School of Medicine of Northwestern University, Chicago, Illinois, USA

**Keywords:** Cardiofaciocutaneous syndrome, craniosynostosis, infantile, Noonan syndrome, prenatal, RASopathy

## Abstract

Fetuses with RASopathies can have a wide variety of anomalies including increased nuchal translucency, hydrops fetalis, and structural anomalies (typically cardiac and renal). There are few reports that describe prenatal-onset craniosynostosis in association with a RASopathy diagnosis. We present clinical and molecular characteristics of five individuals with RASopathy and craniosynostosis. Two were diagnosed with craniosynostosis prenatally, 1 was diagnosed as a neonate, and 2 had evidence of craniosynostosis noted as neonates without formal diagnosis until later. Two of these individuals have Noonan syndrome (*PTPN11* and *KRAS* variants) and three individuals have Cardiofaciocutaneous syndrome (*KRAS* variants). Three individuals had single suture synostosis and two had multiple suture involvement. The most common sutures involved were sagittal (*n* = 3), followed by coronal (*n* = 3), and lambdoid (*n* = 2) sutures. This case series confirms craniosynostosis as one of the prenatal findings in individuals with RASopathies and emphasizes the importance of considering a RASopathy diagnosis in fetuses with multiple anomalies in combination with craniosynostosis.

## 1 | INTRODUCTION

The RASopathies are a group of autosomal dominant disorders caused by pathogenic variants in genes encoding proteins of the RAS/mitogen-activated protein kinase (MAPK) pathway, leading to its dysregulation as the common pathologic mechanism (Tartaglia & Gelb, 2010). These diseases are phenotypically and genetically related, and include Noonan syndrome (NS), Noonan syndrome with multiple lentigines (NSML), Costello syndrome (CS), Cardiofaciocutaneous (CFC) syndrome, Neurofibromatosis type 1 (NF1) and Legius syndrome among others (Rauen, 2013). Genes involved in this pathway include *PTPN11*, *SOS1*, *RAF1*, *BRAF*, *HRAS*, *NRAS*, *CBL*, *MAP2K1*, *MAP2K2*, *NF1*, *RIT1*, *SPRED1*, *SHOC2*, and *KRAS*, as well as several more recently identified genes *LZTR1*, *MRAS*, *PPP1CB*, *SOS2*, *RASA2*, *RRAS*, *RRAS2*, *SYNGAP1*, *SPRED2*, and *MAPK1T* (Gripp et al., 2016; Hebron et al., 2022; Motta et al., 2021; Tartaglia et al., 2022; Tidyman & Rauen, 2016; Viskochil, 2011).

RASopathies are estimated to have a prevalence of 1:1000 live births and exhibit some overlapping phenotypic features such as characteristic facial features, short stature, cardiac and vascular involvement, skeletal abnormalities, cutaneous lesions, developmental delay, intellectual disability, and predisposition for certain cancers (Myers et al., 2014; Rauen, 2013; Tartaglia & Gelb, 2010; Viskochil, 2011). Of note, prenatal findings that are concerning for a RASopathy include increased and/or persistent nuchal translucency, hydrops fetalis, cardiac, renal, and lymphatic defects, polyhydramnios, ascites, macrosomia, and macrocephaly (Biard et al., 2019; Mangels et al., 2021; Myers et al., 2014; Sparks et al., 2020; Stuurman et al., 2019). Stuurman et al. specifically recommends that genetic testing for a RASopathy should be considered in prenatal cases with isolated nuchal translucency >5.0 mm or a nuchal translucency >3.5 mm and at least one additional ultrasound anomaly associated with RASopathy (Stuurman et al., 2019).

Craniosynostosis occurs in 1:2500 live births and involves the premature fusion of one or more cranial sutures (Boulet et al., 2008). This leads to abnormal head shape and can also result in other complications such as increased intracranial pressure, hearing and vision problems, and developmental delay (Boulet et al., 2008; Cunningham et al., 2007). Surgical intervention can mitigate these symptoms, although surgery is not always necessary for mild cases (Johnson & Wilkie, 2011). Although craniosynostosis is considered nonsyndromic in approximately 85% of cases, it occurs in association with other systemic anomalies in about 15%–30% of cases (Johnson & Wilkie, 2011; Kimonis et al., 2007; Timberlake & Persing, 2018). More than 150 syndromic forms of craniosynostosis have been described with genetic and clinical heterogeneity. Syndromic craniosynostosis is more likely to involve multiple sutures or bilateral coronal sutures (Johnson & Wilkie, 2011). The most common genes associated with syndromic craniosynostosis are the fibroblast growth factor receptor genes (*FGFR1*, *FGFR2*, *FGRFR3*), *TWIST1*, and *EFNB1* (Jezela-Stanek & Krajewska-Walasek, 2013).

Although craniosynostosis is an unusual finding among individuals with RASopathies, more cases are being reported (Addissie et al., 2015; Brasil et al., 2012; Davis et al., 2019; Kratz et al., 2009; Lo et al., 2009; Nagai et al., 2021; Rodriguez et al., 2019; Takenouchi et al., 2014; Weaver et al., 2022; Zenker, 2022). The relationship between craniosynostosis and the underlying diagnosis needs to be further characterized to improve early recognition, diagnosis, and intervention. The aim of this study is to expand on current knowledge of craniosynostosis in individuals with a RASopathy. Specifically, this study assesses and describes the phenotypic and genetic heterogeneity of this group of conditions and highlights craniosynostosis as a recurrent prenatal finding in multiple individuals with a RASopathy diagnosis.

## 2 | MATERIALS AND METHODS

### 2.1 | Editorial policies and ethical considerations

This research study was approved by the Cincinnati Children’s Hospital Medical Center (CCHMC) Institutional Review Board. Parental consent was obtained for use of photographs.

### 2.2 | Study design and data collection

This is a retrospective chart review that includes data available from 2010 through 2022. Individuals were included in the study if they had both a clinical diagnosis of craniosynostosis (confirmed by imaging) and a molecularly confirmed RASopathy diagnosis. Five individuals were identified through the RASopathies Program at Cincinnati Children’s Hospital. A query of electronic medical records in I2B2 using ICD 10 codes for Noonan syndrome (ICD 10 Q87.19), CFC syndrome (Q87.89), and craniosynostosis/craniosynostosis repair (Q75.0) did not reveal additional individuals.

Clinical data was abstracted from medical records and entered in a secure database for analysis using de-identified information. Demographic information (e.g., age and sex), phenotypic information (e.g., prenatal findings, extent and location of craniosynostosis, other health problems), radiology images/reports, and genetic test results were collected.

## 3 | RESULTS

Five individuals with confirmed molecular diagnosis of a RASopathy were identified to have craniosynostosis. Two of these participants have Noonan syndrome with pathogenic variants in *PTPN11* (*n* = 1) and *KRAS* (*n* = 1). The remaining three individuals have a diagnosis of CFC syndrome with pathogenic variants in *KRAS* (*n* = 3). Clinical imaging and molecular characteristics of all individuals are reviewed in Table 1.

**FIGURE 1:**
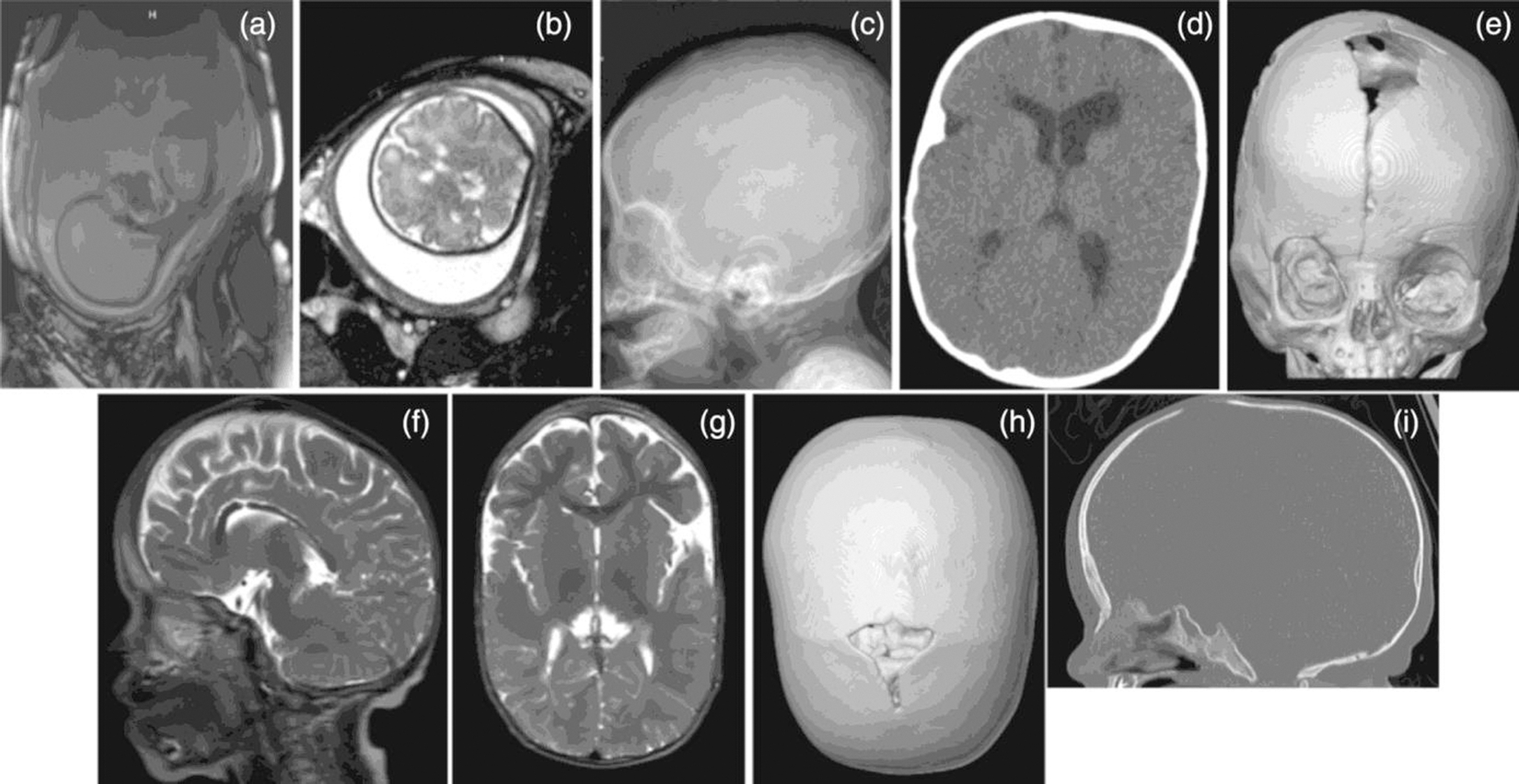
Imaging demonstrating craniosynostosis in Individuals 1–5. (a, b, Individual 1) (a) sagittal view on prenatal MRI; (b) transverse view on prenatal MRI. (c, Individual 2) (c) sagittal view on x-ray. (d, e, Individual 3) (d) transverse view on CT; (e) coronal view of a model of skull. (f, g, Individual 4) (f) sagittal view on MRI; (g) transverse view on MRI. (h, i, Individual 5) (h) CT scan with 3D reconstruction with near complete fusion of sagittal suture, premature closure of lambdoid, coronal, and squamosal sutures; frontal bossing; anterior fontanelle remains patent; (i) frontal bossing, j-shaped sella.

Individual 1 was a male infant diagnosed prenatally with multiple congenital anomalies including asymmetric lambdoid suture initially detected on fetal MRI at 34 weeks 2 days gestation (Figure 1a,b). Additional anomalies detected prenatally were right lung agenesis, tracheoesophageal fistula, and possible right congenital diaphragmatic hernia versus large eventration. Individual 1 was born at 35 4/7 weeks gestation, the fourth child to a 36-year-old mother. Birth weight was 1.72 kg (0.4% percentile) and head circumference was 31.3 cm (2.6% percentile). Apgar scores were 6 and 9 at 1 and 5 min, respectively. A head ultrasound at Day of Life (DOL) 1 found a small cyst in the right choroid plexus, as well as an ovoid cystic area in the suprasellar region. Mild facial dysmorphism was appreciated including prominent forehead, mildly short palpebral fissures, low-set ears, and widely spaced nipples with short sternum. An atrial septal defect was also seen in postnatal echocardiogram. Computer tomography (CT) scan at 1 month of age confirmed right lambdoid craniosynostosis. He underwent an endoscopic craniectomy for his craniosynostosis around 10 weeks of age. At 3 months of age, a mixed left subdural and subarachnoid hemorrhage was noted on a head ultrasound, as well as mild third and lateral ventriculomegaly. Following a complicated medical and surgical course, he died at 3 months of age of respiratory failure due to pulmonary hemorrhage and pulmonary hypertension. Postmortem whole exome sequencing identified a de novo pathogenic variant in *PTPN11* (c.802G>T, p.Gly268Cys). These results confirmed a diagnosis of Noonan Syndrome.

Individual 2 is a 7-year-old female born at 35 weeks gestation after a pregnancy complicated by polyhydramnios and limited prenatal care. She weighed 2.89 kg (15.6% percentile). After birth, she had a 23 day neonatal intensive care unit (NICU) admission at local hospital due to lung immaturity, feeding issues, hyperbilirubinemia requiring phototherapy and failure to thrive. Physical exam was significant for dolichocephaly and microcephaly, turricephaly, micrognathia, coarse facial appearance, proptosis, furrowed brow, hypertelorism with down-slanting palpebral fissures, epicanthal folds, short bulbous nose with prominent philtrum, narrow high arched palate, and low-set posteriorly rotated ears. The hands had deep palmar and plantar creases with persistence of fetal fat pads and feet had hyperkeratosis. She had generalized xerosis, and sparse hair. Sagittal craniosynostosis was confirmed with x-rays (Figure 1c). Echocardiogram identified coarctation of the aorta and pulmonary stenosis. Other health concerns include renal tubular acidosis, significant postnatal growth retardation, and developmental delays. She had a craniotomy for her craniosynostosis at 2 months of age. A brain MRI at 1 year of age found symmetric cytotoxic edema involving optic radiations, posterior pons/medulla, and inferior anterior basal ganglia. No ventriculomegaly was observed. She had multiple admissions for failure to thrive during first year of life. She was initially admitted at this institution at 11 months old for failure to thrive and Genetics was consulted. An expanded RASopathy panel identified a previously reported pathogenic missense variant in *KRAS* (c.101C>G, p.Pro34Arg).

Individual 3 is now a 7 years old female. Prenatal history is notable for ultrasounds concerning for bicoronal craniosynostosis. She was born at 39 weeks 6 days gestation, weighing 3.34 kg (45.2% percentile). She was the first child to a 28-year-old mother. At birth she was intubated for respiratory distress and was admitted to the NICU; she was extubated 6 h later. At DOL 1, echocardiogram noted hypertrophic cardiomyopathy and ultrasound of head noted sagittal synostosis. Additional follow up for abnormal head shape (dolichocephaly, prominent sagittal ridge with open and posterior fontanelles, constricted temporoparietal bones giving an incomplete cloverleaf appearance) included a CT scan with 3D reconstruction which identified right coronal and sagittal suture synostosis (Figure 1d,e). She remained in the NICU for 3 weeks due to feeding intolerance and cardiac concerns. Her facial features included bilateral shallow orbits, low-set ears, mid-face retrusion, deviated septum with ridged nasal tip, and a short neck. Genetics was consulted during NICU stay and microarray, craniosynostosis panel, and RASopathy panel were ordered. Microarray and craniosynostosis panel resulted negative. RASopathy panel identified pathogenic missense variant in *KRAS* gene (c.173C>T, p.Thr58Ile). She had an endoscopic craniectomy for her craniosynostosis at 1 month old. An MRI when the individual was 10 months old showed aqueductal stenosis, lateral and third ventriculomegaly, and a subdural hematoma.

Individual 4 is an 11-year-old male born at 33 weeks gestation after pregnancy complicated by hydrops fetalis and polyhydramnios. His birthweight was 3.45 kg (43.3% percentile). He was intubated at delivery for respiratory distress and remained intubated for 3 weeks. He stayed in the NICU for 10 weeks due to chronic lung disease, bilateral subdural hemorrhages, sinus tachycardia, and pulmonary hypertension. He was re-admitted to the hospital at 15 months old due to respiratory distress, hydrocephalus, ventriculitis, and cardiomyopathy. Genetics was consulted and individual was noted to have coarse facial features, dolichocephaly with prominent sagittal ridge, and dry skin. Craniofacial features included down-slanting palpebral fissures, bilateral epicanthal folds, depressed nasal bridge, anteverted nares, low-set cupped ears, coarse eyebrows, facial hypotonia, square facial shape, wide mouth with downturned corners and hypertelorism. A brain MRI at 15 months of age demonstrated moderate enlargement of the lateral and third ventricles was observed. This brain MRI also indicated that individual 4 had congenital hydrocephalus secondary to aqueductal stenosis, previously treated with ventricular drainage. CT scan noted incidental sinus and temporal bone findings in addition to a new small left frontal subdural hematoma. Additionally, his medical history was notable for hypertrophic cardiomyopathy with mild left ventricular outlet tract obstruction and dysplastic aortic, mitral and tricuspid valves (Figure 1f,g). Noonan spectrum chip testing was ordered and identified a pathogenic missense variant in *KRAS* (c.178G>C, p.Gly60Arg). He followed up with Genetics at 5 year old and was noted to have prominent sagittal suture concerning for craniosynostosis. He did not have further imaging or surgical intervention for repair of craniosynostosis.

Individual 5 is a 3-year-old female born at 37 weeks 2 days gestation and weighed 3.54 kg (61.4% percentile), the third child of a 33 year old mother. Prenatal history noted possible renal concern on 20-week ultrasound but no further follow up was done during pregnancy. After birth, she was admitted to the NICU for 6 weeks due initially to meconium aspiration. During NICU stay, this individual also had abnormal echo noting a PFO, brain MRI which resulted unremarkable, modified barium swallow study showing delayed laryngeal closure and aspiration of breast milk, had a g-tube placed, and had an abdominal ultrasound which was unremarkable. Most recent echo now notes mild concentric left ventricular hypertrophy, mild hypoplastic transverse arch, and possible bicuspid valve. Sleep study showed obstructive sleep apnea; now post adenoidectomy. Skeletal survey resulted unremarkable. She also has short stature (1st percentile), hypotonia and hyperflexibility requiring use of SMOs, oral aversion, reflux, and nystagmus. On physical exam, she is noted to have distinct features including bilateral epicanthal folds, down-slanting palpebral fissures, coarse facial features, low-set ears with thick lobes and dimple in posterior side of each lobe. Also, she is noted to have always had a very large anterior fontanelle although it has finally begun closing and a hump is now developing at the anterior edge of the fontanelle. Genetics work-up included microarray, skeletal dysplasia panel, *SNIP1* sequencing, and lysosomal storage disorder analysis. All resulted negative. Around 15 months old, exome sequencing was ordered and resulted with a de novo pathogenic variant in *KRAS* (c.178G>C, p.-Gly60Arg). Of note, a maternally inherited likely pathogenic variant in MT-ATP6 (m.8717A>G) was also identified with approximately 2% heteroplasmy. After diagnosis, she followed up with Genetics to discuss further management. A CT of head with 3D reconstruction was ordered and noted mild to moderate third ventricular enlargement and premature closure of multiple cranial sutures (Figure 1h,i).

## 4 | DISCUSSION

This study describes five individuals with RASopathies and co-occurring craniosynostosis. Two individuals had abnormal head shape concerning for craniosynostosis noted prenatally (Table 1). These results are consistent with other recent case reports of individuals with RASopathies and craniosynostosis (Ueda et al., 2017; Weaver et al., 2022) adding to the growing literature that demonstrates craniosynostosis can occur in individuals with RASopathies.

Craniosynostosis has been described in individuals with pathogenic variants in other genes of the RAS/MAPK pathway including *BRAF* (Davis et al., 2019; Ueda et al., 2017), *KRAS* (Addissie et al., 2015; Brasil et al., 2012; Kratz et al., 2009; Lo et al., 2009; Schubbert et al., 2006; Ueda et al., 2017), *PPP1CB* (Bertola et al., 2017), *SHOC2* (Takenouchi et al., 2014), *PTPN11* (Davis et al., 2019; McDonald et al., 2018; Ueda et al., 2017), *RAF1* (Rodriguez et al., 2019), and *HRAS* (Nagai et al., 2021; Weaver et al., 2022).

In addition to these genes, recent studies have demonstrated that pathogenic variants in *ERF*, a molecule that interacts with the RAS/MAPK pathway, can cause craniosynostosis (ERF-related craniosynostosis) (Twigg et al., 2013). In studies of mice carrying a mutation within this gene, they initially showed delayed markers of osteogenesis and later craniosynostosis (Twigg et al., 2013; Twigg & Wilkie, 2015). A reduced dosage of ERF protein leading to craniosynostosis may suggest an interaction between the RAS and the FGFR signaling pathways as multiple independent authors have shown (Kim et al., 2003; Shukla et al., 2007). However, the role of ERF activation for the development of craniosynostosis is still not well understood and more studies are needed to elucidate these mechanisms.

All the individuals in this cohort exhibit typical features of RASopathies in addition to cranial deformities. However, three of the five individuals were noted to have abnormal head shape concerning for craniosynostosis prenatally or shortly after birth (Table 1). This early onset has not been described in prior reports of RASopathy-associated craniosynostosis. The majority of these individuals also had multi-organ involvement such as cardiac abnormalities and feeding concerns.

Three of these individuals had involvement of the sagittal sutures, two had involvement of the lambdoidal sutures, and three had involvement of the coronal sutures (Table 1). Other studies regarding RASopathies and craniosynostosis have found little involvement of coronal suture (Ueda et al., 2017). Three of these five individuals required surgical repair for the craniosynostosis. A few of these individuals also exhibit additional uncommon complications such as right lung agenesis and tracheoesophageal fistula (Individual 1), and aqueductal stenosis (Individuals 3 and 4). These results highlight the evolving phenotypic associations in the RASopathies.

A limitation of this retrospective study is that not all these individuals received exome sequencing so we cannot rule out the possibility of additional diagnoses related to the craniosynostosis. For the two individuals with exome trio sequencing data available there was no secondary diagnosis associated with craniosynostosis. This finding combined with the recently reported cases of craniosynostosis and RASopathies suggests a true association. Several individuals have limited information about prenatal medical history and limited imaging available. This is a single center experience and additional larger cohort studies will be important to define the spectrum of manifestations related to this underrecognized complication of the RASopathies.

Our results add to the growing clinical evidence that craniosynostosis is related to RAS activation. In the setting of a RASopathy diagnosis, there is likely increased risk for presentation of craniosynostosis. Among this group of disorders, craniosynostosis appears to be consistently associated with Noonan, CFC, and Costello syndromes with pathogenic variants most frequently reported in *BRAF*, *HRAS*, *KRAS*, and *PTPN11* (Table 2) (Ueda et al., 2017; Weaver et al., 2022; Zollino et al., 2017). More research is needed to determine how often craniosynostosis occurs in individuals with RASopathies, and what other factors may influence this relationship. These results further illustrate that when an individual presents with craniosynostosis, RASopathies should be included in the differential, especially when other abnormalities are present (e.g., congenital heart defects, aqueductal stenosis). Likewise, when an individual presents with a RASopathy, clinicians should monitor for craniosynostosis as a possible complication. Recognizing craniosynostosis as a possible manifestation of RASopathies could decrease time to diagnosis through informing testing utility. Future research could be directed toward gathering more quantitative information on craniosynostosis and RASopathies, specifically the incidence and prevalence in larger cohorts.

## Figures and Tables

**TABLE 1:** Description of patients with craniosynotosis and RASopathy.

Reference	Sex at birth	Age at craniosynostosis dx	Age at RASopathy diagnosis	Clinical diagnosis	Gene	Codon change	Mutation type	Amino acid change	Mode of inheritance	Predicted functional effect	Craniosynostosis	Diagnostic imaging	Perinatal description

This report (1)^a^	M	Prenatal 34 w2 d	Postmortem d. 3 months	NS	*PTPN11*	c.802G>T	Missense	P.G268C	De novo	Pathogenic	R Lamboidal	CT	Diaphragmatic hernia dx 23 wl d, right lung agenesis, cardiac abnormalities
This report (2)^a^	F	Birth	11 m	CFC	*KRAS*	c.101C>G	Missense	P.P34R	Unknown	Pathogenic	Bilateral coronal	CT	Polyhydramnios, prematurity, respiratory distress
This report (3)^a^	F	Prenatal	2 m	NS	*KRAS*	c.173C>T	Missense	P.T58I	De novo	Pathogenic	R Coronal; Sagittal	CT	Intubation, feeding intolerance
This report (4)	M	1 y	1 y	CFC	*KRAS*	c.178G>C	Missense	p.G6R	Unknown	Pathogenic	Sagittal	CT	Hydrops fetalis, polyhydramnios, premature, intubation, chronic lung disease, bilateral subdural hemorrhages, tachycardia, pulmonary hypertension
This report (5)	F	2 y	3 y	CFC	*KRAS*	c.178G>C	Missense	P.G60R	De novo	Pathogenic	Sagittal, Lamboidal, coronal	CT	Cardiac abnormalities, feeding intolerance, obstructive sleep apnea
Davis 2019	M	3 y6 m	Unspecified, after craniosynostosis dx	NS	*PTPNU*	c.188A>G	Missense	P.Y63C	Unknown	Pathogenic	Sagittal	CT	Cardiac abnormalities, Horseshoe kidney, submucous cleft palate
Ueda 2017	F	9 y	1 m	NS	*PTPN11*	c.922A>G	Missense	P.N308D	Unknown	Pathogenic	Sagittal	CT	Feeding difficulties, cardiac abnormalities
Ueda 2017	M	5 y	4 m	NS	*PTPNU*	c.922A>G	Missense	P.N308D	Unknown	Pathogenic	Sagittal	CT	No concerns noted
Ueda 2017	M	7y	5 y	NS	*PTPNU*	c.1471C>G	Missense	P.P491A	Unknown	Pathogenic	Sagittal	CT	No concerns noted
McDonald 2018	M	6y	14 y	NSML	*PTPNU*	c.1492C>T	Missense	P.R498W	De novo	Pathogenic	Sagittal	CT	Cardiac abnormalities
Addissie 2015	F	2 m	Unspecified, after craniosynostosis dx	NS	*KRAS*	c.101C>A	Missense	P.P34Q	De novo	Pathogenic	Sagittal	CT	Increased nuchal translucency
Kratz 2009^a^	M	Neonatal	Neonate	NS	*KRAS*	c.173C>T	Missense	P.T58I	De novo	Pathogenic	Sagittal, bilateral coronal	CT	Polyhydramnios, cystic hygroma
Kratz 2009	M	6 m	8 m	NS	*KRAS*	c.178G>A	Missense	P.G60S	De novo	Pathogenic	L Lamboidal	CT	Polyhydramnios
Ueda 2017	F	4y11 m	Neonate	CFC	*KRAS*	c.178G>C	Missense	P.G60R	Unknown	Pathogenic	Sagittal, Metopic, Coronal, Lamboidal	CT	Fetal hydrops, extreme prematurity, cardiac abnormalities
Brasil 2012	M	6 m	Neonate	NS	*KRAS*	c.214A>C	Missense	P.M72L	Maternal	Pathogenic	Metopic	CT	Increased nuchal translucency
Davis 2019	M	5 m	Unspecified, after craniosynostosis dx	NS	*KRAS*	c.40G>A	Missense	P.V14I	Unknown	Pathogenic	Sagittal	CT	Cryptorchidism, Macrocephaly, GERD, cardiac abnormalities, bilateral arachnoid cyst
Lo 2009	M	Unk	9 y6 m	NS	*KRAS*	c.40G>A	Missense	P.V14I	De novo	Pathogenic	Sagittal	CT	No concerns noted
Schubbert 2006	F	1 y2 m	Unknown	NS	*KRAS*	c.173C>T	Missense	P.T58I	De novo	Pathogenic	Sagittal	CT	Cardiac abnormalities
Ueda 2017	M	17 y	1 y6 m	CFC	*KRAS*	c.178G>C	Missense	P.G60R	Unknown	Pathogenic	Sagittal, Metopic, Coronal, Lamboidal	CT	No concerns noted
Rodriguez 2019^a^	F	2 w	11 y2 m	NSML	*RAF1*	c.788T >G	Missense	P.V263G	Not paternal	Likely Pathogenic	“Cloverleaf”	CT	Polyhydramnios
Takenouchi 2014	M	2 y3 m	Unspecified, after craniosynostosis dx	NS	*SHOC2*	c.4A>G	Missense	p.S2G	De novo	Pathogenic	Sagittal, R Coronal, Bilateral Lamboidal	CT	Left pleural effusion dx 18 w, fetal hydrops
Davis 2019	M	5 m	Unspecified, after craniosynostosis dx	NS	*BRAF*	c.735A>T	Missense	P.L245F	Unknown	Pathogenic	Sagittal	CT	Prematurity, failure to thrive, cardiac abnormalities, pyloric stenosis
Ueda 2017	M	3 y5 m	11 m	CFC	*BRAF*	c.755G>C	Missense	P.R252P	Unknown	Pathogenic	Sagittal	CT	Prematurity
Ueda 2017	F	4y8 m	9 m	CFC	*BRAF*	c.770A>G	Missense	P.Q257R	Unknown	Pathogenic	Sagittal	CT	No concerns noted
Davis 2019	M	2 y5 m	Unspecified, after craniosynostosis dx	CFC	*BRAF*	c.1497A>C	Missense	P.K499N	De novo	Pathogenic	Sagittal	CT	Prematurity, cardiac abnormalities, poor weight gain
Davis 2019	F	4y6 m	17 m	CFC	*BRAF*	c.1741A>G	Missense	P.N581D	Unknown	Pathogenic	Sagittal	CT	Cardiac abnormalities, hepatomegaly
Ueda 2017	F	12 y	10 y	CFC	*BRAF*	c.1796C>G	Missense	P.T599R	Unknown	Pathogenic	R Squamous	CT	Feeding difficulties
Ueda 2017	F	3 y6 m	2 y3 m	CFC	*BRAF*	c.1802A>T	Missense	P.K601I	Unknown	Pathogenic	Sagittal, Lamboidal	CT	No concerns noted
Bertela 2017	M	3 m	12 y	NSLAH	*PPP1CB*	c.146C>G	Missense	P.P49R	Unknown	Pathogenic	Sagittal, bilateral partial coronal	CT	No concerns noted

Abbreviations: CFC, Cardiofaciocutaneous syndrome; CT, computer tomography scan; NS, Noonan syndrome; NSLAH, Noonan Syndrome-Like Disorder with Loose Anagen Hair.

a Cranionsynostosis diagnosis in perinatal period.

**TABLE 2:** Summary of RASopathy genes, type of craniosynostosis, and diagnosis in perinatal period.

Gene	Total	Sagittal	Lamboid	Squamous	Metopic	Coronal	Multiple	Concern noted prenatally	Concern noted as neonate

BRAF	7	5		1 (Right)			1		
HRAS	6	3			1		2		
KRAS	13	5	1 (Left)		1	1	5	1	2
PPP1CB	1						1		
PTPN11	6	5	1 (Right)					1	
RAF1	1						1		1
SHOC2	1						1		
Total	35	18	2	1	2	1	11	2	3
% total		55%	6%	3%	6%	3%	33%	6%	9%

## Data Availability

The data that support the findings of this study are available on request from the corresponding author. The data are not publicly available due to privacy or ethical restrictions.
